# Conventional and organic soil management as divergent drivers of resident and active fractions of major soil food web constituents

**DOI:** 10.1038/s41598-019-49854-y

**Published:** 2019-09-18

**Authors:** Paula Harkes, Afnan K. A. Suleiman, Sven J. J. van den Elsen, Johannes J. de Haan, Martijn Holterman, Eiko E. Kuramae, Johannes Helder

**Affiliations:** 1 Laboratory of Nematology, Dept. Plant Sciences, Wageningen University, Droevendaalsesteeg 1, 6708 PB Wageningen, The Netherlands; 2 Department Microbial Ecology, Droevendaalsesteeg 10, 6708 PB Wageningen, The Netherlands; 3 Wageningen University & Research Open Teelten, Edelhertweg 10, Lelystad, The Netherlands; 4 KWR Watercycle Research Institute, Groningenhaven 7, 3433 PE Nieuwegein, The Netherlands

**Keywords:** Agroecology, Microbial ecology

## Abstract

Conventional agricultural production systems, typified by large inputs of mineral fertilizers and pesticides, reduce soil biodiversity and may negatively affect ecosystem services such as carbon fixation, nutrient cycling and disease suppressiveness. Organic soil management is thought to contribute to a more diverse and stable soil food web, but data detailing this effect are sparse and fragmented. We set out to map both the resident (rDNA) and the active (rRNA) fractions of bacterial, fungal, protozoan and metazoan communities under various soil management regimes in two distinct soil types with barley as the main crop. Contrasts between resident and active communities explained 22%, 14%, 21% and 25% of the variance within the bacterial, fungal, protozoan, and metazoan communities. As the active fractions of organismal groups define the actual ecological functioning of soils, our findings underline the relevance of characterizing both resident and active pools. All four major organismal groups were affected by soil management (p < 0.01), and most taxa showed both an increased presence and an enlarged activity under the organic regime. Hence, a prolonged organic soil management not only impacts the primary decomposers, bacteria and fungi, but also major representatives of the next trophic level, protists and metazoa.

## Introduction

Intensification of food and feed production is often accompanied by increased nutrient inputs, intense pesticide applications, frequent tillage, and irrigation management^1^. The negative environmental implications of these practices include eutrophication, increased salinization, soil erosion and biodiversity loss^2,3^. Our knowledge of the consequences of agricultural intensification on belowground biodiversity is still limited and fragmented. It has been reported that larger-sized soil biota are more negatively affected by high-input agricultural practices than soil microbes^4^, but is it widely acknowledged that more detailed studies are required to map the effects of soil management on soil microbiota^5^.

Soils harbour a quarter of the world’s biodiversity and reside among the most complex habitats on earth^6,7^. Soil biota plays a role in many essential soil functions such as nutrient cycling, carbon and water retention, soil texture formation and the interaction with the plant community^8^. Most intensive interactions between microbes and plants take place in the rhizosphere, where the plant is able to select and boost a subset of the microbial community by the release of rhizodeposits - a broad range of carbon-containing substances (e.g. root cells, mucilage, volatiles and exudates). The composition of the rhizobiome, the subset of the soil biota present in the rhizosphere, is co-determined by plant identity and age^9–13^. With the advent of affordable high throughput DNA sequencing techniques, the impact of plants on the identity and density of rhizosphere inhabitants can be mapped. Insight in this interaction could help to design soil management measures promoting a rhizobiome that would optimally support plant growth and improve crop yield^14^.

As in many other habitats, most soil inhabitants have to cope with unpredictable food availability^15^. In order to survive periods of food scarcity, various microorganisms can reversibly reduce their metabolic activity over an extended period of time^16^. Such a condition is referred to as a state of dormancy^17^. In bulk soil, typically 80% of the cells and 50% of the operational taxonomical units (OTU’s) are dormant. This so-called “microbial seedbank”^18^ is alert in the sense that it can detect and respond to environmental stimuli (e.g. organic substrates) that are associated with favourable growing conditions^19^. Plant roots produce and release a broad spectrum of environmental stimuli and, as such, the rhizosphere is a hotspot of microbial activity^20,21^.

Given the typically high percentage of dormant microbiota in soil, it is relevant to discriminate between the resident and the active microbial community when considering soil ecological processes. The resident community refers here to all organisms present in a certain spatial unit of soil, whereas the active community comprises the fraction of the resident community that is metabolically active. Ribosomal (r)RNA is considered a representation of the active microbial community, while rDNA characterises the total microbial community^22,23^. Hence, combined profiling of community rDNA and rRNA will provide insight in both aspects of local microbial assemblies. More specifically, such a characterisation will provide information about microbial fractions, whose activity is positively or negatively affected by any kind of external influence. Although a number of soil ecological studies considered both the active and the resident microbial community^24–26^, large scale mapping of shifts in the active soil microbiome has been hampered so far by the low throughput nature and the costs of currently available kits for RNA extraction from soil. A combination of elements from various published protocols allowed us to develop a fast and affordable method for nucleic acid extraction from soil.

The aim of this study was to investigate the long-term impact of soil management regimes (including conventional and organic soil management) on resident (rDNA) and active (rRNA) microbial communities. To this end, we collected bulk and rhizosphere samples in two different growth stages of summer barley (*Hordeum vulgare*) grown on two distinct soil types - peaty and sandy soil - under the various types of soil management. Four major organismal groups were assessed: bacteria and fungi - representing the primary decomposers - and protists and nematodes - two major grazers on the bacterial and fungal communities. Specific variable ribosomal DNA regions were selected for the characterization of each of the four organismal groups. We hypothesise that (i) prolonged exposure to distinct soil management regimes will impact both the resident and active fractions of the primary decomposers (bacterial and fungal community), (ii) shifts in the primary decomposer community due to soil management will be translated into associated changes in the active fractions of major representatives of the next trophic level (protists and metazoa), (iii) exposure to rhizosphere will have a stronger stimulating effect on the active fractions of the primary decomposers (*i.e*. bacteria and fungi) than on protists and metazoa.

## Results

### Essentials on the characterisation of soil biota

Nucleic acids (total DNA and RNA) from 104 bulk soil and rhizosphere samples were extracted using a novel, lab-made protocol (Table 1). Contrary to bacteria, fungi and protists, the 2 g soil subsamples will not provide a proper representation of the metazoan community. We included metazoa in this study because this fraction of the soil microfauna was co-extracted with the microbial community, and therefore probably in close physical contact with this community. MiSeq sequencing of organismal group-specific 16S (bacteria) or 18S (fungi, protists, and metazoa) ribosomal DNA and cDNA fragments resulted in ≈ 31 million reads (15.5 million forward and 15.5 million reverse), and on average ≈ 75,000 reads per sample. After filtering, a total of 8,297,203 sequences were retained comprehending 724 samples for all taxa together. Comprehensive sampling of the microbial community was obtained for all treatments, with average sequence coverage of 63%, 70%, 96% and 97% for respectively bacteria, protozoa, fungi and metazoan as determined by Good’s coverage estimator (Supplementary Table S3).

**Table 1:** Lab-made protocol for the direct extraction of DNA and RNA from soil developed by combining elements from several soil nucleic acid extraction methods^79,80,94^.

Step	Procedure
1.	Weigh 2 g of thoroughly mixed soil, transfer it to a 15 mL bead tube), and add 1.5 g of coarse silicon carbide powder (46 grit). (Keep bead tubes on ice from step 1–9).
2.	Add 2.5 mL of bead solution (181 mM disodium phosphate, 121 mM guanidinium thiocyanate), 0.25 mL of lysis buffer (150 mM NaCl, 4% (w/v) SDS, 0.5 M Tris), and 0.8 mL of a 120 mM ammonium aluminum sulfate dodecahydrate solution^79^.
3.	Add 3.5 mL of phenol:chloroform:isoamyl alcohol (25:24:1, pH: 8.0, 4 °C) and mix it manually to disintegrate the biphasic layer.
4.	Place the bead tubes for 10 minutes on a Digital Vortex genie 2 (SI-A256) with a SI-H512 horizontal 15 ml tube holder at maximum speed (2,850 rpm) at 4 °C^80^. Note: place no more than 4 tubes in the tube holder as this would lower the rpm.
5.	Incubate the tubes horizontally at −20 °C for 10 minutes. Thereafter: repeat step 4, and continue to step 6.
6.	Centrifuge the tubes for 10 minutes (2,500 × g) at 4 °C to separate the soil particles from the lysate.
7.	Transfer 3 mL of the upper aqueous phase to a new 15 mL tube, and add 1.5 mL of ice-cold precipitation solution (an aqueous solution of 5 M NaCl, 22 mM citric acid anhydrous salt, and 29 mM trisodium citrate dihydrate)
8.	Centrifuge the tubes for 10 minutes (2,500 × g) at 4 °C to separate the precipitate from the nucleic acids.
9.	Transfer 4 mL of supernatant to a new 15 mL tube containing 5 mL of isopropanol at room temperature (RT). Mix gently by hand for 5 s and centrifuge the tubes for 15 minutes (2,500 × g) at 4 °C to precipitate the nucleic acids.
10.	Discard the isopropanol, and air dry the pellet for 5 minutes at RT.
11.	Add 1 mL binding solution (at RT) to the pellet (binding solution: an aqueous solution of 5 M guanidinium thiocyanate and 30 mM Tris-HCl (pH: 6.5) with 9% (v/v) isopropanol), and vortex to re-dissolve the pellet.
12.	Load 0.5 mL of solution to a silica spin filter (RP20 CommaPrep RNA extraction column, Biocomma, China) to bind the nucleic acids (DNA and RNA)^94^, and spin for 30 s at 10,000 g.
13.	Discard the flow-through, add 0.75 mL of washing solution (10 mM Tris-HCl, pH: 6.5), 100 mM NaCl, and absolute EtOH final v/v 50%) to the spin filter, spin for 30 s at 10,000 g. Repeat this step 3 times.
14.	Air-dry the filter for 5 minutes, and transfer the spin filter to a clean collection tube.
15.	Add 0.2 mL of elution buffer (10 mM Tris-HCl, pH: 8.0) to the spin filter, and spin for 30 s at 10,000 g.
16.	Collect the eluate (0.2 mL) and store it at −80 °C until further use.

This protocol was optimized for nucleic acid extraction from sandy soils with a range of organic matter contents.

### Contrasts between resident versus active fractions of four major soil food web constituents

PERMANOVA identified Nuclei Acid (*i.e*. rRNA and rDNA) as the main factor responsible for the differences between samples for all four organismal groups (Table 2). The factor explained 14 to 25% of the overall variance (P < 0.01). The second important explanatory factor of the observed variation was Location (6 to 13%), which is assumed to be attributable mainly to differences in soil type. ‘Vredepeel’ is characterized by sandy soils, whereas peaty soil typifies ‘Valthermond’. For the primary decomposers, a higher percentage of the overall variation was explained by soil type (11% and 13% for bacteria and fungi, respectively) when compared to the representatives of the next trophic level (8 and 6% for protists and metazoans, respectively) (for all groups P < 0.01).

**Table 2:** PERMANOVA analysis was used to test the effect of a number of variables on the composition of bacterial, fungal, protozoan and metazoan assemblages.

Source	F	R2	P
**Bacteria**			
Nucleic Acid	67.659	0.219	9.99-05
Location	32.604	0.106	9.99-05
Treatment	5.465	0.053	9.99-05
Sample Type	7.158	0.023	9.99-05
Time Point	3.700	0.012	0.00240
Residuals		0.410	
**Fungi**			
Nucleic Acid	48.045	0.144	9.99-05
Location	43.315	0.130	9.99-05
Treatment	11.086	0.100	9.99-05
Sample Type	7.174	0.022	9.99-05
Time Point	6.923	0.021	9.99-05
Residuals		0.410	
**Protozoa**			
Nucleic Acid	73.162	0.208	9.99-05
Location	27.792	0.079	9.99-05
Treatment	6.344	0.041	9.99-05
Sample Type	7.550	0.022	9.99-05
Time Point	4.788	0.018	9.99-05
Residuals		0.461	
**Metazoa**			
Nucleic Acid	76.172	0.245	9.99-05
Treatment	6.014	0.058	9.99-05
Location	17.300	0.056	9.99-05
Sample Type	12.655	0.041	9.99-05
Time Point	6.981	0.022	9.99-05
Residuals		0.429	

The following factor were displayed: Nucleic Acid (cDNA/DNA), Location (Vredepeel/Valthermond), Treatment (ConSlu, ConMin, Org (Vredepeel), Comp and No-Comp (Valthermond)), Sample type (Bulk/Rhizosphere) and Time point (Vegetative/Generative) as factors. Differences are considered significant if P < 0.01. P = probability associated with the Pseudo F statistic.

UniFrac, a method that uses phylogenetic distances as a measure for the comparison of microbial communities^27^, was used to verify the impact of individual variables. Both weighted and unweighted UniFrac demonstrated that all variables that were shown to have a significant effect on the bacterial community. This was in essence also true for fungi, although Sample Type was not significant in case of unweighted UniFrac (P = 0.069). For Protozoa, only the effect of treatment was not significant while using unweighted UniFrac (P = 0.097). In case of metazoa, the only non-significant variable was Time Point for the unweighted UniFrac (P = 0,084) (see Supplementary Table 4).

**Figure 1:**
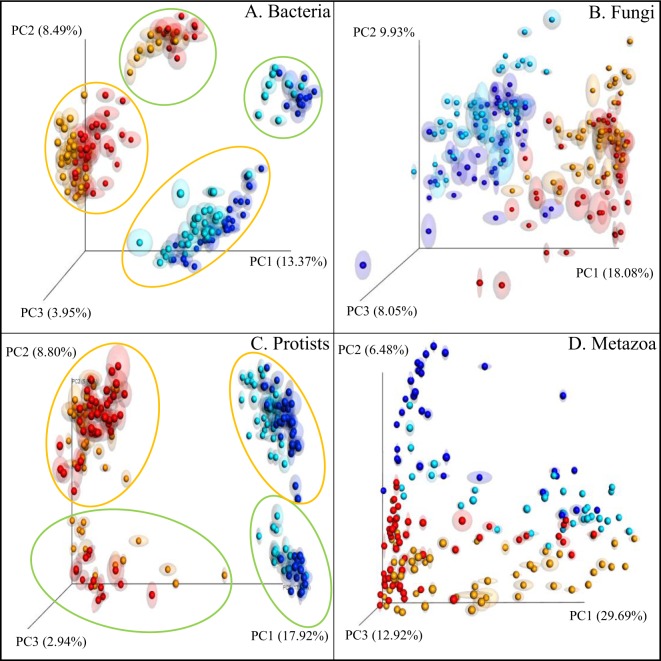
Principal coordinate analysis (PCoA) ordination of a Bray-Curtis dissimilarity matrix. Plots illustrate distances between communities (104 soil samples; for each sample both the resident (rDNA) and the active (rRNA) community were characterized) for each organismal group. (**A**) Bacteria; (**B**) Fungi; (**C**) Protozoa, and (**D**) Metazoa. Colours were used to distinguish between rRNA-bulk, rRNA-rhizosphere (ochre), and rDNA-bulk (dark blue), rDNA-rhizosphere (light blue). Locations are indicated by an ochre circle (Vredepeel, sandy soil) or a green circle (Valthermond, peaty soil).

In addition to the PERMANOVA, we generated a principal coordinate analysis (PCoA) ordination of a Bray-Curtis dissimilarity matrix (Fig. 1). There is a clear differentiation visible between active (rRNA) and resident communities (rDNA). The resident communities cluster (blue and light blue) and are separated from the active microbial communities (red and ochre) for all four organismal groups. This separation between clusters was most obvious for Bacteria (Fig. 1A) and Protists (Fig. 1C). For Fungi (Fig. 1B) and Metazoans (Fig. 1D) this separation between active and resident communities was visible as well, but less pronounced.

Visualisation of the significant location effects that were demonstrated by the PERMANOVA for all four organismal groups resulted in two distinct patterns. Whereas a complete separation of clusters was observed for bacteria and protists (encircled in ochre and green in Fig. 1A,C), a less clear separation was seen for fungal and metazoan communities (Fig. 1B,D). It appeared to be difficult to visualize the impact of all variables for the four organismal groups with a single set of graphic settings. In Supplementary Fig. S3 alternative settings were used to picture the location effect for fungi and metazoans.

Exposure to rhizosphere conditions resulted in a difference between both the resident and the active microbial community. For bacteria, the difference in community composition between bulk and rhizosphere soil is reflected at both rDNA and rRNA level (blue and light blue, and red and ochre, respectively) (Fig. 1A). For protists, differences between bulk and rhizosphere soil were most pronounced at rDNA level (Fig. 1C). At rRNA level, protist communities only showed differentiation in peaty soils (location Valthermond). It is noted that the PERMANOVA pinpointed significant rhizosphere effects for all four organismal groups (Table 2).

### Microbial taxa contributing to contrasts between resident and active communities

**Figure 2:**
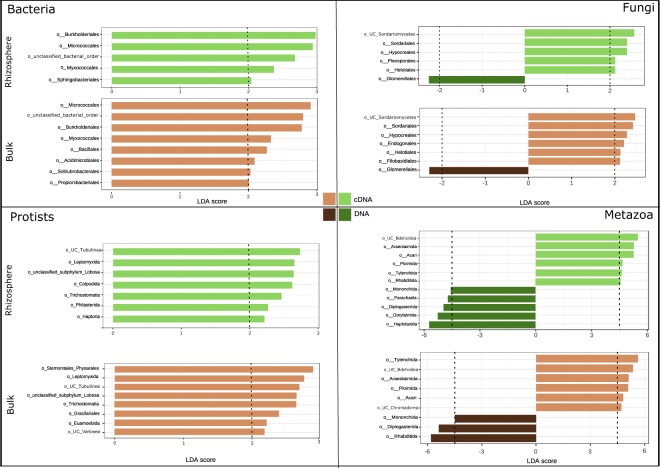
LEfSe analysis of bacterial, fungal, protists and metazoan OTUs identifying taxa for which a major part of the population was active in rhizosphere (light green) or in bulk soil (light brown) (LDA score >2), or for which a major part of the population is dormant in rhizosphere (green) or in bulk soil (brown) (LDA scores <−2).

To pinpoint taxon-specific differences between resident and active communities and different soil management, we further analysed the samples of location ‘Vredepeel’. Based on LEfSe with LDA thresholds for discriminative features set at ≥2 or ≤−2, a total of 9 bacterial, 8 fungal, 11 protozoan, and 12 metazoan orders contributed significantly to the differences between the resident (rDNA-based) and active (rRNA-based) communities (Fig. 2)

Four bacterial orders were identified as highly active in both rhizosphere and bulk soil (Fig. 2A). For barley rhizosphere the active Sphingobacteriales were distinct, whereas Bacillales, Acidimicrobiales, Solirubrobacterales and Propionibacteriales were predominately found in bulk soil. It is noted that only Propionibacteriales showed a large contrast between bulk and rhizosphere samples, the other three bacterial orders had LDA scores just below 2 in rhizosphere soils (data not shown).

Analysis of the fungal community revealed four orders that were highly active in both bulk and rhizosphere soil. The order Glomerellales was present (DNA) but barely active in both soil compartments (LDA score below -2). The bulk soil was typified by active members of the orders Filobasidiales and Endogonales. Notably, the contrast between bulk soil and rhizosphere was subtle for Filobasidiales, and more substantial for members of the Endogonales (data not shown).

Protists, being a predominantly bacterivorous group in soil, were included as major representatives of the next trophic level. Colpodida, Philasterida and Haptoria were identified as protist orders with an enhanced metabolic activity in the barley rhizosphere.

Our analyses revealed active rotifers, mites, nematodes, and insects in the rhizosphere compartment. A pronounced difference (LDA distance of 8) between the activity of the bacterivorous nematode order Rhabditida in bulk and rhizosphere soil. Rhabditida are known as extreme opportunists^28^. This ecological characteristic is reflected in Fig. 2.

### Effects of soil management regimes on community structures

**Figure 3:**
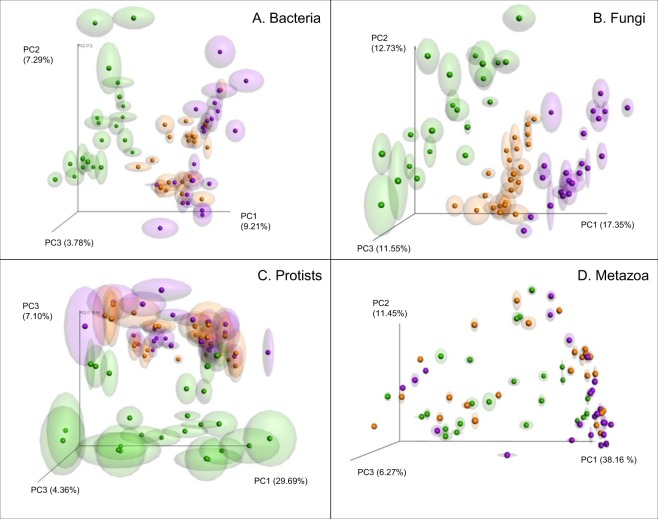
Principal coordinate analysis (PCoA) ordination of a Bray-Curtis dissimilarity matrix. Plots illustrating distances between the active fractions of communities at location Vredepeel (sandy soil) (n = 72) for (**A**) Bacteria, (**B**) Fungi, (**C**) Protozoa and (**D**) Metazoa. Colors were used to indicate soil management regimes: ConMin (purple), ConSlu (orange), and Organic (green).

Based on our analysis, compost treatment at location ‘Valthermond’ had no significant impact on any of the analysed organismal groups (see Supplementary Table S5). In contrast, at location ‘Vredepeel’ the three soil management regimes (ConMin, ConSlu and Org) showed to have a significant effect on the microbial community structure. Both the PERMANOVA and a PCoA (Table 3, Fig. 3) indicated a distinct microbial community structure for Org fields as compared to communities found under ConMin and ConSlu management. This soil management effect was most evident for the active communities (rRNA) of Bacteria, Fungi and Protozoa. Results were verified with weighted and unweighted UniFrac. For both UniFrac variants the effect of soil management (‘Treatment’) was significant for all four organismal groups (Supplementary Table S6). A similar analysis on the resident community (rDNA) revealed a comparable, though less pronounced pattern (Supplementary Fig. S4). For Metazoans, no clear soil treatment effect was observed in both the active and the resident communities.

**Table 3:** PERMANOVA analysis was used to test the effect of the following factors: Nucleic Acid (cDNA/DNA), Sample Type (bulk soil/rhizosphere), Treatment (soil management regime: ConMin, ConSlu, or Org), and Time Point (vegetative and generative).

Source	F	R2	P
**Bacteria**			
Nucleic Acid	92.223	0.35373	9.99-05
Sample Type	7.158	0.02745	0.0001
Treatment	9.906	0.07599	9.99-05
Time Point	7.588	0.02911	0.0004
Residuals		0.40	
**Fungi**			
Nucleic Acid	56.589	0.22513	9.99-05
Sample Type	8.448	0.03361	9.99-05
Treatment	18.436	0.14669	9.99-05
Time Point	12.776	0.05083	9.99-05
Residuals		0.40	
**Protozoa**			
Nucleic Acid	120.58	0.39706	9.99-05
Sample Type	6.588	0.02169	0.0014
Treatment	5.922	0.039	0.0002
Time Point	13.585	0.04473	1.00E-04
Residuals		0.389	
**Metazoa**			
Nucleic Acid	41.323	0.19827	9.99-05
Sample Type	13.602	0.06526	9.99-05
Treatment	9.311	0.08935	9.99-05
Time Point	9.06	0.04347	9.99-05
Residuals		0.50	

Differences are considered significant if P < 0.01. P = probability associated with the Pseudo F statistic.

#### Bacteria

In general, prolonged organic soil management on sandy soil boosted the abundance of almost all bacterial orders rDNA level (Supplementary Fig. S5.1b). Out of the 38 bacterial taxa that significantly differed in abundance between ConMin and Org fields, 36 taxa were more abundant in the organic treatment. When considering the active fraction of the bacterial community, 47 taxa were significantly affected by soil management, of which 31 taxa were found to be more active in Org. Among the soil management-affected taxa, 16 showed a higher activity in ConMin fields.

**Figure 4:**
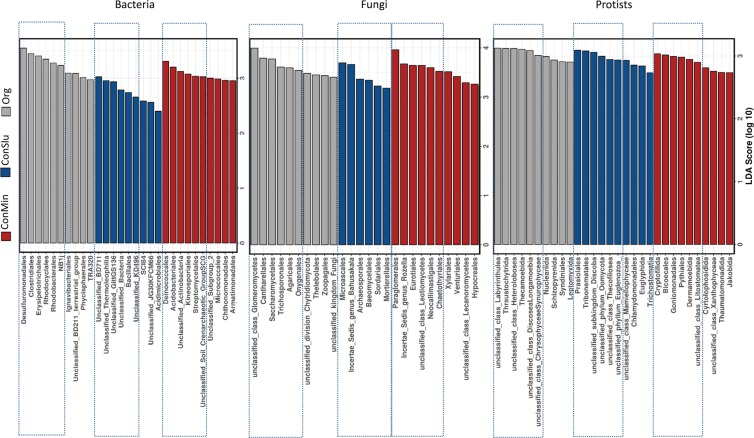
Discriminant active bacterial, fungal, and protozoan taxa indicated by LEfSe analysis resulting from distinct soil management types at location Vredepeel: ConMin (red), ConSlu (blue) and Org (grey). For each treatment and organismal group, six taxa with the highest LDA scores are delineated.

As compared to two conventional soil treatments ConMin and ConSlu, prolonged organic soil management has boosted the activity (rRNA) of a range of bacterial orders. Desulfuromonadales, Clostridiales, Erysipelotrichales, Rhodocyclales, and Rhodobacterales had the highest LDA scores (LEfSe, LDA score >2, Fig. 4), and their increased activity was confirmed by ANOVA (grey arrows in Supplementary Fig. S5.1). The orders Bacillales, Deinococcales, Micrococcales, Acidobacteriales, Kineosporiales, and Streptomycetales were identified as indicators for conventional soil treatments. ANOVA did not confirm the status of the order Kineosporiales as indicator taxon for the ConMin treatment (red arrow 4 in Supplementary Fig. S5.1). It is noted that no further attention was paid to bacterial taxa without a formal systematic name.

#### Fungi

Out of the 30 fungal taxa that significantly differed in OTU abundance (rDNA), 22 taxa were more abundant in organic fields compared to conventional managed fields (Supplementary Fig. S5.2). When concentrating on the fungal orders of which the activity (rRNA) was affected by soil management type, 19 taxa were more active in organic soils and 10 were promoted under conventional soil management regimes (ConMin and ConSlu) (Supplementary Fig. S5.2).

LEfSe analysis of fungal rRNA sequences revealed the orders Glomeromycetes (unclassified class), Cantharellales, Saccharomycetales, Trichosporonales, Agaricales, and Onygenales as indicative for the organic regime (Fig. 4). In most cases, ConSlu-promoted taxa were also activated under the ConMin regime. The order Microascales was identified as a specific indicator for ConSlu-treated fields. ANOVA confirmed the indicator status of this order for the ConSlu treatment (blue arrow 1 in Supplementary Fig. S5.2). The orders Paraglomerales, Eurotiales, Neocallimastigales, and Chaetothyriales were identified as indicators for conventional farming system using mineral fertilizer only.

#### Protozoa

The total abundance (rDNA) of 28 protist taxa was upregulated in organic treatment, while 13 taxa were promoted under conventional soil management (Supplementary Fig. S5.3). The impact of organic soil management at rRNA level showed an opposite trend since only 12 out of 41 taxa showed a higher OTU abundance in the fields under organic management.

Thraustochytrida and Thecamoebida were identified as protist indicators for organic soil management. Unclassified members of the classes Labyrinthulea and Heterolobosea were characterized by higher densities and higher activity in Org fields (Fig. 4., Supplementary Fig. S5.3). Prasiolales, Tribonematales, Cryptofilida, Phytiales, Dermamoebida and Bicoecales were identified as indicator taxa for conventional soil management (ConMin and ConSlu) (Fig. 4). Notably, ANOVA did not confirm the indicator status of Tribonematales and Bicoecales (Supplementary Fig. S5.3).

#### Metazoa

The impact of the three soil managements regimes had little impact on the metazoan communities. Only a few orders were characterized as indicators by LEfSe analysis. Mononchida, an order of predatory nematodes, and the mollusc superfamily Nuculoidea were more actively abundant under organic farming. The nematode orders Dorylaimida and Areaolaimida were more active under the ConSlu regime, while Tylenchida and Monhysterida were indicative for ConMin. ANOVA gave non-corresponding results for a number of the aforementioned orders.

## Discussion

Characterisation of both resident and active fractions of bacterial and fungal communities as well as their primary consumers, protists and metazoans, generated a holistic view on long-term effects of various soil management systems including organic farming. Analysis of 104 samples from fields with barley as main crop underlines the relevance of distinguishing active (rRNA) and resident (rDNA) communities. For all four organismal groups nucleic acid type was the most important explanatory variable. The long-term impact of soil management was significant for all four organismal groups and dozens of taxa were identified that showed an increased presence and an enlarged activity under the organic soil management regime.

A few aspects of the use of rRNA and rDNA as markers for active and resident soil biota require further attention. Ribosomal RNA is a highly abundant transcript, and rDNA is a multi-copy gene for which the number of copies varies enormously between organismal groups. Among bacteria, the number of rRNA operons is moderately diverse; typically, bacterial genes harbour 1 to 15 copies^29^. For protists, the number of rDNA copies is substantially higher. Focussing on a range of diatoms and dinoflagellates, individual species were shown to harbour rDNA copy number in the range of 100 to 10,000^30^. For fungal taxa, a recent study estimated the number of rDNA copies to range between 14 and 1,400. This variation in fungal rDNA copy number could not be linked to trophic preferences or other easily observable ecological characteristics^31^. Based on copy number variation, it is clear that rRNA and rDNA data from microbial communities should not be used for quantitative comparisons between organismal groups, neither should it be used for comparison of abundances or activities between high taxonomic level taxa within an organismal group. In this study, we infer rRNA to represent the active community. Nevertheless, dormant soil inhabitants may have ribosomes with functional rRNAs^32^ to allow organisms to resume activity as soon as condition are favourable again. However, it is reasonable to assume that the transition from a dormant to a physiologically active state will be accompanied by a substantial increase in rRNA level. Although it is appreciated that the use of rRNA/rDNA data for the characterisation of active and resident microbial communities in soil has a number of inherent constraints, within-taxon comparison of rDNA-based sequence data under various environmental conditions is likely to reveal robust and valuable information on the impact of these conditions.

Community characterisation by either of the two types of nucleic acids revealed that dormancy was a phenomenon relevant for all four organismal groups under investigation.

### Bacteria

Several bacterial orders were shown to be active in both bulk soil and in the rhizosphere. Sphingobacteriales, as an exception, was indicated as specifically active in the rhizosphere (Fig. 2). Pfeiffer *et al*.^33^ found a similar enrichment of this order in the rhizosphere of maize. A study by Haichar *et al*.^34^ showed Sphingobacteriales accumulation on roots of wheat, rape and barrel clover. This apparent general rhizosphere accruement is in line with the observed upregulated activity near the roots of barley. Propionibacteriales were identified as being specifically active in bulk soil. Propionibacteriales are known to contribute to both primary and secondary fermentations^35^. The observed activity of Propionibacteriales in bulk soil could therefore relate to the distinct fertilisation regimes.

### Fungi

Both in bulk soil and in the rhizosphere representatives of the orders of Sordariales, Hypocreales, Helotiales were highly active (all belonging to the Ascomycota). This in accordance with a survey on four conventional arable fields in Austria that revealed the dominant presence of the same three fungal orders^36^. However, the high abundance of these Ascomycete orders is not specific for arable soils, Tedersoo *et al*.^37^ found similar orders associated with tree roots. Both these studies indicate high abundance (based on DNA data) of the three orders and our data demonstrates that these fungal orders are also highly active. Glomerellales (Ascomycota) showed a negative LDA score for bulk as well as rhizosphere, indicating dormancy. Drought has been shown to specifically decrease protein abundances of Glomerellales^38^. As sampling took place during a relatively dry period, this could explain the observed dormancy. Unfortunately, little is known about the ecology of Glomerellales.

### Protozoa

Barley rhizosphere was characterized by active representatives of the orders of Haptoria, Colpodida and Philasterida. Haptoria are ubiquitous free-living predatory ciliates in soils^39^, whereas Colpodida are predominantly grazers of bacteria^40^. The high bacterial density in the rhizosphere could explain the accumulation of active Colpodida. Philasterida are known to occur in terrestrial habitats, but additional information about their ecology is scarce. Bulk soil was typified by active Euamoebida, a supergroup that has been indicated active in grassland and forest mineral soils^41^. In the same study, the paraphyletic class Variosea was found to be a dominant active class in bulk soil collected from grassland. Our study confirms the presence of active members of Variosea in bulk soil from barley fields. This class was defined only recently^42^, and little is known about their ecological role in soil. Physarales is suggested to play an important role in litter breakdown^43^ and therefore it is no surprise that they are specifically active in bulk soils.

### Metazoa

As compared to bacteria, fungi and protists, metazoa showed the strongest compartment effect (R^2^ = 0.041, Table 3 (Metazoa / Sample type)). This might point at a large difference in community composition between bulk soil and rhizosphere keeping in mind that the subsamples analysed in this study were too small to be representative for each of the compartments. Nevertheless, this result is in line with the highly density and activity of soil micro fauna in the immediate vicinity of plant roots that has been well reported for a range of systems^44–46^. Our analysis indicated also a number of orders as dormant (LDA <−2). This category included the orders Haplotaxida (oligochaetes), Dorylaimida (nematodes) and Parachaela (tardigrades). For Haplotaxida and Dorylaimida the observed signals may have originated from unhatched eggs. A number of plant parasites reside within the order Dorylaimida, and these may remain unhatched until signals from a suitable host plant are perceived. Tardigrades including the Parachaela member *Hypsibius dujardini* are known for their ability to survive relatively dry conditions such as in the upper soil layers at the time of sample collection in a dormant state^47^. In case of bulk soil, the dormancy of the nematode order Rhabditida was most prominent. This is not unexpected as members of the Rhabditidae are highly opportunistic bacterivores that enter the dormant Dauer stage under unfavourable conditions (e.g. drought, food scarcity)^48,49^. It is noted that due the relatively large size of soil metazoa, environmental DNA could have contributed to relatively low RNA to DNA ratio which could erroneously be interpreted as a signal for dormancy^50^.

The effect of organic soil management on bacterial communities is relatively well documented^51–54^. To the best of our knowledge there are no other studies investigating the effects of distinct soil management regimes on four organismal groups simultaneously.

### Bacteria

At DNA level, ANOVA revealed that 44 out of the 48 bacterial taxa were more abundant under organic management (Supplementary Fig. 5.1). This observation is in line with earlier research on the effects of organic soil management^55^. However, at activity level (as revealed by rRNA-based analysis) ‘only’ 1/3 of the 50 bacterial taxa were most active under organic soil management conditions (Supplementary Fig. 5.1). As microbial activity matters in terms of soil food web functioning, these results underline the relevance of taking – next to abundance data – activity data into account.

The following bacterial orders contributed most to the difference between organic *versus* conventional soil management regimes (in terms of OTU abundance and LEfSe score): Desulfuromonadales (δ-proteobacteria), Clostridiales (Firmicutes), Rhodocyclales (β-proteobacteria), Rhodobacterales (α-proteobacteria). Desulfuromonadales, an order typifying Org fields, harbour a range of sulphate and sulphur reducing bacteria^56^. Their enhanced activity could relate to the slightly higher S content of the Org fields (Org:247 mg S/kg soil, ConMin and ConSlu: 193 and 214 mg S/kg)^57,58^. Clostridiales are metabolically diverse but increased abundance of members of this order has been observed upon the addition of organic matter with a high recalcitrant C content^59^. The Org fields studied here received crop residues as green manure, and hard-to-degrade plant remains could have promoted the Clostridiales. Rhodocyclales and Rhodobacterales may act as denitrifiers under low oxygen conditions^60,61^, but the background of their activation under the Org regime remains unclear.

Conventional fields were characterized by highly active Actinobacteria (Kineosporiales, Streptomycetales and Micrococcales). This was also observed in previous research on the impact of conventional and organic cropping systems on the microbial community. In a survey over 3 years, Orr *et al*.^62^ detected a similar increase in Actinobacteria, but they also showed a strong sample year effect. For the interpretation of our data this should be taken into consideration.

### Fungi

In total 70% of the significantly soil management-affected fungal orders were more abundant under the organic regime. In general, this higher abundance was accompanied by higher activity. The majority of the fungal OTUs were assigned to the Ascomycota. Among the Ascomycota, there are numerous decomposers of organic substrates (such as leaf litter, wood, and manure) and more studies reported them as the major fungal phylum present in agro-ecosystems^63,64^. Two Basidiomycetes (Agaricales, Cantharellales) were found to be abundant in organic fields. Both orders harbour numerous wood and litter decomposer taxa^65^, substrates that are added to these fields in relatively large quantities. Onygenales were rarely found in the conventional fields but highly active in the organic soils. This order is associated with animal dung^66,67^. So, active members of the Onygenales are probably the result of the application of cattle manure in the Org fields.

The strongest fungal indicator for organic farming was an “unclassified class of Glomeromycetes’. Glomeromycetes are known to form arbuscular mycorrhizas and colonize the roots of vascular land plants including barley^68^. In a recent study on the same experimental farm (Vredepeel), AM fungi were also found to be more abundant under organic soil management^57,58^. AM fungi can stimulate the decomposition of recalcitrant organic matter, and makes nitrogen bioavailable^69^. Hence, the distinct type of manure used under organic management might explain the specific stimulation of Glomeromycetes.

Paraglomerales, an order of the Glomeromycetes was predominantly found in conventional systems. This finding was corroborated by Dai *et al*.^70^ who found *Paraglomus* to be positively associated with the conventional production of wheat. The relation between *Paraglomerales* and fertilization system would require further investigation.

### Protozoa

In contrast to the primary decomposers, organic soil management decreased the activity of many protozoa. In parallel, we observed an increase in total abundance (rDNA) under the organic regime for a majority of the soil management-affected taxa. Increased densities of protozoa as a result of organic amendments have been reported before^71^. Under controlled greenhouse conditions, application of organic fertilizers increased bacterivorous and omnivorous protists, and strongly reduced the relative abundance of plant pathogenic protists^72^. We aren’t aware of other studies on the impact of organic amendment to soil protist activity.

### Metazoa

Organic soil management stimulated the activity an order of predatory nematodes Mononchida, and members of the mollusc order Nuculoidea. Predatory Mononchida feed on other nematodes, but this does not hold for all life stages. Larval stages are too small to capture other nematodes, and they feed on bacteria^73^. The strongly enriched bacterial community under the organic regimes may have promoted the activity of the Mononchida. The impact of soil management on metazoa will not be discussed further, as the numbers of individuals present in the 2 g soil samples are relatively low (with some nematode taxa as an exception). Hence, sampling effects could easily obscure soil management effects.

Our results demonstrate that prolonged (>15 consecutive years) exposure to distinct soil management regimes causes shifts in the primary decomposer assemblies (bacteria and fungi) as well as changes in primary consumer communities (protists and metazoa). It is concluded that organic management practices results soil microbial communities that are demonstrable distinct from the communities under the conventional regimes. However, our fragmentary knowledge about the ecology and food preference of soil microbiota limited our ability to link most of the observed shifts to desired soil-bound ecosystem services.

## Methods

### Study sites

Samples were collected from barley fields at two locations in The Netherlands: (1) WUR experimental farm ‘Vredepeel’ is located in the southeast of the Netherlands (51°32 N and 5°51E) and is characterized by sandy soil (93,3% sand, 4.5% silt, 2.2% clay) and an organic matter (OM) content of 3–5%. Three different soil management strategies were applied from 2001 onwards: ConMin, ConSlu and Org. ConMin fields solely received mineral fertilizer and processed organic fertilizer without organic matter (liquid mineral concentrates), and ConSlu fields were supplemented with mineral fertilizer and pig/cow slurry. In case of organic soil management, farmyard manure and cow slurry were applied, and no pesticides were used. For further details of the set up and layout of the soil management experiments see additional research reports^74–77^. (2) WUR experimental farm in Valthermond is situated in the northeast of the Netherlands (52°50′N, 6°55′E) and characterized by sandy peat soil (90% sand, 7% silt, 3% clay) and a high OM content (up to 14%). At Valthermond, the effect of the application of compost was investigated (yearly application of 15 tons (green) compost per hectare) since 2013. For this study, we made a comparison between the control and compost plots.

#### Soil sampling

At both experimental farms, barley (*Hordeum vulgare*) is one of the main crops in the crop rotation system. Due to a slight latitudinal difference, development of the barley plants in ‘Valthermond’ was one week delayed as compared to ‘Vredepeel’. Samples were collected at two time points in spring 2017 (during the vegetative and the generative stage of the crop, see also Supplementary Table S1).

At the ‘Vredepeel’, each of the three fields was divided in 6 subfields of 540 m^2^ (Supplementary Fig. S1). In each subfield, a bulk soil and a rhizosphere sample were collected. Rhizosphere composite samples were taken by harvesting all barley plants within a vicinity of 20 × 20 cm. Excessive soil was removed from the root system by shaking the plants. Immediately thereafter, the plants were transferred to a field laboratory, and rhizosphere samples were collected by brushing off soil that adhered to the roots from 10 individual barley plants. For bulk soil, three cores were collected between the barley rows using an auger (ø1.5 cm, depth approximately: 15 cm), and thoroughly mixed in pre-labelled bags.

In total 36 composite samples (18 rhizosphere and 18 bulk) were taken at each time point. Rhizosphere soil and bulk soil samples were frozen immediately after sampling in liquid nitrogen and transported on dry ice to the laboratory and stored at −80 °C.

At the ‘Valthermond’ location, samples were taken in the first 2 meters of the subfield. In total 4 subfields of each treatment (Supplementary Fig. S2) were sampled resulting in 16 samples (8 rhizosphere and 8 bulk) at each time point. Barley rhizosphere samples were collected as described above. Hence, a total of 104 soil samples (72 from ‘Vredepeel’, 36 from ‘Valthermond’) were used for further analysis.

### DNA/RNA extraction and cDNA synthesis

Both DNA and RNA were simultaneously extracted from soil samples, using a lab-made protocol based on a combination of published protocols. This protocol uses a subsample of 2 g from a thoroughly mixed soil sample as starting point (Table 1). Contrary to bacteria, fungi and protozoa, a subsample of 2 g will not give a proper representation of the metazoan community^78^. Metazoan DNAs were co-extracted with DNAs from other microbiota, and the resulting data represent microfauna that was presumably living in the close physical vicinity of the observed microbiota. For this reason, metazoa were included in this study.

After precipitation of humic substances from the 2 g subsample by the chemical flocculant NH_4_ Al (SO_4_)_2_^79^, and the removal of proteins by a standard phenol:chloroform:isoamyl alcohol mixture, samples were physically disrupted by bead beating using a Vortex Genie 2 with tubes attached to a horizontal tube holder. After a standard isopropanol precipitation, nuclei acids were re-dissolved in a binding solution. DNA and RNA were further purified using a silica-based RP20 CommaPrep RNA extraction column^80^ (see Table 1 for technical details). Quality and quantity of the obtained RNA and DNA was measured with a Nanodrop and Qubit. Until further processing, nucleic acid eluates were stored in −80 °C.

For synthesis of cDNA from extracted RNA the Maxima First Strand cDNA Synthesis Kit for RT-qPCR (Fermentas, Thermo Fisher Scientific Inc., USA) was used according to the manufacturer’s instructions. All individual DNA and cDNA samples were diluted to 1 ng/ul and 0.1 ng/ul respectively, to serve as a template for PCR amplification.

### PCR amplification and sequencing

For the characterisation of bacterial, fungal, protist and metazoan communities, variable regions (V) of 16S or 18S ribosomal DNA were amplified. For bacteria, the V4 region was amplified, while for protozoa, fungi and metazoa respectively the V9, V7-V8, V5-V7 regions were targeted (for details see Table 4). To prepare the samples for Illumina sequencing, a two-step PCR procedure was followed. In the first PCR, locus-specific primers extended with an Illumina read area and the appropriate adapter (Table 4) were used to produce primary amplicons. Three µl of diluted DNA or cDNA template was used with the following temperature profile: 3 min 95 °C, followed by 35 × (95 °C, 10 s; 55 °C, 20 s; 72 °C, 20 s) and a final extension step of 72 °C of 5 min. This was done in triplicate for all samples and for each of the four organismal groups. The second PCR step was performed on 40x diluted amplicons of PCR step 1. This PCR 2 was conducted to attach the Illumina index and the Illumina sequencing adaptor (3 min 95 °C, followed by 10 × (95 °C, 10 s; 60 °C, 30 s; 72 °C, 30 s) and a final extension step of 72 °C of 5 min). Products of PCR 1 and 2 were randomly checked on gel to ensure amplification was successful. Finally, all PCR products were pooled and sent for sequencing (Bioscience, Wageningen Research, Wageningen, The Netherlands) using the Illumina MiSeq Desktop Sequencer (2*300nt paired-end sequencing) according to the standard protocols.

**Table 4:** PCR1 primers with adaptor sequences (underlined), read area, and locus-specific part (bold).

Target	Primer	Adaptor	Target region	Reference
Bacteria	515 F	TCGTCGGCAGCGTCAGATGTGTATAAGAGACAG**GTGCCAGCMGCCGCGGTA**	V4	^95^
Bacteria	806 R	GTCTCGTGGGCTCGGAGATGTGTATAAGAGACAG**GGACTACHVGGGTWTCTAAT**		^90^
Fungi	FF390.1	TCGTCGGCAGCGTCAGATGTGTATAAGAGACAG**CGWTAACGAACGAGACCT**	V7-8	^96^
Fungi	FR1	GTCTCGTGGGCTCGGAGATGTGTATAAGAGACAG**AICCATTCAATCGGTAIT**		^97^
Protozoa	1391 F	TCGTCGGCAGCGTCAGATGTGTATAAGAGACAG**GTACACACCGCCCGTC**	V9	^98^
Protozoa	EukBr	GTCTCGTGGGCTCGGAGATGTGTATAAGAGACAG**TGATCCTTCTGCAGGTTCACCTAC**		^99^
Meta	M1041F	TCGTCGGCAGCGTCAGATGTGTATAAGAGACAG**AGAGGTGAAATTCTTGGAYCGY**	V5-7	^100^
Meta	M1648R	GTCTCGTGGGCTCGGAGATGTGTATAAGAGACAG**ACATCTAAGGGCATCACAGAC**		^100^

### Bioinformatics pipeline

The composition of microbial communities of the soil samples was analysed based on the sequencing data obtained from the Illumina MiSeq platform. Reads were first sorted into the experimental samples according to their index combination. Thereafter, they were sorted into the four organismal groups based on their locus-specific primer sequences (general run statistics can be found in Supplementary Table S2).

Sequences were processed with Hydra pipeline version 1.3.3^81^ implemented in Snakemake^82^. Forward and reverse reads were paired only for bacteria and fungi while single-end sequences were analysed for protozoa and metazoa. The four taxonomical groups were quality trimmed by BBDUK and then merged via VSEARCH^83,84^. Resulting unique sequences were then clustered at 97% similarity by using the usearch_global method implemented in VSEARCH and a representative consensus sequence per *de novo* OTU was determined^84^. The clustering algorithm also performs chimera filtering to discard likely chimeric OTUs with UCHIME algorithm in *de novo* mode^85^ implemented in VSEARCH. Sequences that passed quality filtering were then mapped to a set of representative consensus sequences to generate an OTU abundance table. Representative OTU sequences were assigned to a taxonomic classification via BLAST against the Silva database (version 12.8) for bacteria, fungi and metazoa, and against the Protist Ribosomal Reference database PR2^86^ for protozoa using SINA^87^. Sequences belonging to chloroplasts, cyanobacteria and mitochondria were discarded from the bacterial dataset, and sequences not belonging to Fungi and Metazoa were removed from the 18S Fungi and Metazoa datasets. The Protozoa dataset was filtered for Streptophyta, Metazoa, fungal and unclassified Opisthokonta sequences. Low-abundance OTUs (those with abundance of <0.005% in the total data set) were discarded prior to analysis^88^. Samples were transformed using Hellingers’ transformation for all downstream analyses.

### Statistical analyses

Sampling effort was estimated by Good’s coverage^89^. For statistical analysis, we explored β diversity patterns by performing principal coordinate analysis (PCoA) with Bray-Curtis dissimilarity using QIIME software^90^. Permutational multivariate analysis of variance (PERMANOVA) was used to compare the microbial community structure between soil managements taken from different sites and with different plant growth stages for active and resident community for 4 different taxa. This was performed with 999 permutations using the adonis function, based on Bray-Curtis and UniFrac (weighted and unweighted) distances using the “vegan” package^91^ in R. To investigate the indicator taxa involved in the differences between resident and active community, a linear discriminate analysis (LDA) effect size (LEfSe) was conducted in Microbiome Analyst^92^ to explore the differential microbial populations at the family level for the four different taxa^93^. A significance level of α ≤ 0.05 was used for all biomarkers evaluated in this study.

## Supplementary information

Supplementary Tables and Figures

Supplementary Table S3

## Data Availability

The raw sequences were submitted to the NCBI Sequence Read Archive (SRA) database under study accession number BioProject ID PRJNA543417.

## References

[1] Tilman D, Balzer C, Hill J, Befort BL (2011). Global food demand and the sustainable intensification of agriculture. Proc. Natl. Acad. Sci..

[2] Foley JA (2005). Global consequences of land use. Science.

[3] Tilman D (2001). Forecasting agriculturally driven global environmental change. Science.

[4] Postma-Blaauw MB, de Goede RGM, Bloem J, Faber JH, Brussaard L (2010). Soil biota community structure and abundance under agricultural intensification and extensification. Ecology.

[5] El Mujtar V, Munoz N, Mc Cormick BP, Pulleman M, Tittonell P (2019). Role and management of soil biodiversity for food security and nutrition; where do we stand?. Glob Food Sec..

[6] Bardgett, R. *The biology of soil: a community and ecosystem approach*. (Oxford University Press, 2005).

[7] Roesch LF (2007). Pyrosequencing enumerates and contrasts soil microbial diversity. ISME J..

[8] Henneron L (2015). Fourteen years of evidence for positive effects of conservation agriculture and organic farming on soil life. Agron Sustain Dev.

[9] Chaparro JM, Badri DV, Vivanco JM (2014). Rhizosphere microbiome assemblage is affected by plant development. ISME J..

[10] Grayston SJ, Wang SQ, Campbell CD, Edwards AC (1998). Selective influence of plant species on microbial diversity in the rhizosphere. Soil Biol Biochem.

[11] Inceoglu O, Abu Al-Soud W, Salles JF, Semenov AV, van Elsas JD (2011). Comparative Analysis of Bacterial Communities in a Potato Field as Determined by Pyrosequencing. PLoS One.

[12] Kuske CR (2002). Comparison of soil bacterial communities in rhizospheres of three plant species and the interspaces in an arid grassland. Appl Environ Microb.

[13] Schlemper TR (2017). Rhizobacterial community structure differences among sorghum cultivars in different growth stages and soils. FEMS Microbiol Ecol.

[14] Knief C (2014). Analysis of plant microbe interactions in the era of next generation sequencing technologies. Front Plant Sci.

[15] Morita, R. Y. *Bacteria in oligotrophic environments: starvation-survival lifestyle*. Vol. 1 (Chapman & Hall New York, 1997).

[16] Blagodatskaya E, Kuzyakov Y (2013). Active microorganisms in soil: Critical review of estimation criteria and approaches. Soil Biol Biochem.

[17] Stevenson LH (1978). Case for Bacterial Dormancy in Aquatic Systems. Microbial Ecol.

[18] Lennon JT, Jones SE (2011). Microbial seed banks: the ecological and evolutionary implications of dormancy. Nat Rev Microbiol.

[19] De Nobili M, Contin M, Mondini C, Brookes PC (2001). Soil microbial biomass is triggered into activity by trace amounts of substrate. Soil Biol Biochem.

[20] Hinsinger P, Bengough AG, Vetterlein D, Young IM (2009). Rhizosphere: biophysics, biogeochemistry and ecological relevance. Plant Soil.

[21] Reinhold-Hurek B, Bunger W, Burbano CS, Sabale M, Hurek T (2015). Roots Shaping Their Microbiome: Global Hotspots for Microbial Activity. Annu Rev Phytopathol.

[22] De Vrieze J (2016). Presence does not imply activity: DNA and RNA patterns differ in response to salt perturbation in anaerobic digestion. Biotechnol Biofuels.

[23] Ofek M, Voronov-Goldman M, Hadar Y, Minz D (2014). Host signature effect on plant root-associated microbiomes revealed through analyses of resident vs. active communities. Environ Microbiol.

[24] Baldrian P (2012). Active and total microbial communities in forest soil are largely different and highly stratified during decomposition. ISME J..

[25] Nunes I (2018). Soil bacteria show different tolerance ranges to an unprecedented disturbance. Biol Fert Soils.

[26] Schostag M (2019). Bacterial and protozoan dynamics upon thawing and freezing of an active layer permafrost soil. ISME J..

[27] Lozupone C, Knight R (2005). UniFrac: A new phylogenetic method for comparing microbial communities. Appl Environ Microb.

[28] Bongers T (1990). The Maturity Index - an Ecological Measure of Environmental Disturbance Based on Nematode Species Composition. Oecologia.

[29] Espejo RT, Plaza N (2018). Multiple Ribosomal RNA Operons in Bacteria; Their Concerted Evolution and Potential Consequences on the Rate of Evolution of Their 16S rRNA. Front Microbiol.

[30] Godhe A (2008). Quantification of Diatom and Dinoflagellate Biomasses in Coastal Marine Seawater Samples by Real-Time PCR. Appl Environ Microb.

[31] Lofgren LA (2018). Genome-based estimates of fungal rDNA copy number variation across phylogenetic scales and ecological lifestyles. Mol Ecol.

[32] Nanamiya H (2010). Bacillus subtilis mutants harbouring a single copy of the rRNA operon exhibit severe defects in growth and sporulation. Microbiology.

[33] Peiffer JA (2013). Diversity and heritability of the maize rhizosphere microbiome under field conditions. Proc. Natl. Acad. Sci..

[34] Haichar FZ (2008). Plant host habitat and root exudates shape soil bacterial community structure. ISME J.

[35] Johnson J, Cummins C (1972). Cell wall composition and deoxyribonucleic acid similarities among the anaerobic coryneforms, classical propionibacteria, and strains of *Arachnia propionica*. J Bacteriol.

[36] Klaubauf S (2010). Molecular diversity of fungal communities in agricultural soils from Lower Austria. Fungal Divers.

[37] Tedersoo L (2009). Ascomycetes associated with ectomycorrhizas: molecular diversity and ecology with particular reference to the Helotiales. Environ Microbiol.

[38] Bastida F (2017). Differential sensitivity of total and active soil microbial communities to drought and forest management. Global Change Biol.

[39] Vďačný P (2014). The Chaos Prevails: Molecular Phylogeny of the Haptoria (Ciliophora, Litostomatea). Protist.

[40] Vďačný P, Foissner W (2018). Re-analysis of the 18S rRNA gene phylogeny of the ciliate class Colpodea. Eur J Protistol.

[41] Geisen S (2015). Metatranscriptomic census of active protists in soils. ISME J..

[42] Cavalier-Smith T, Chao EEY, Oates B (2004). Molecular phylogeny of Amoebozoa and the evolutionary significance of the unikont *Phalansterium*. Eur J Protistol.

[43] Kamono A, Matsumoto J, Kojima H, Fukui M (2009). Characterization of myxomycete communities in soil by reverse transcription polymerase chain reaction (RT-PCR)-based method. Soil Biol Biochem.

[44] Chen X, Liu M, Hu F, Mao X, Li H (2007). Contributions of soil micro-fauna (protozoa and nematodes) to rhizosphere ecological functions. Acta Ecologica Sinica.

[45] Griffiths BS (1994). Microbial-Feeding Nematodes and Protozoa in Soil - Their Effects on Microbial Activity and Nitrogen Mineralization in Decomposition Hotspots and the Rhizosphere. Plant Soil.

[46] Irshad U, Villenave C, Brauman A, Plassard C (2011). Grazing by nematodes on rhizosphere bacteria enhances nitrate and phosphorus availability to *Pinus pinaster* seedlings. Soil Biol Biochem.

[47] Vasanthan T, Nederveen JP, Stone J (2019). Quantum-like decreased embryogenesis time with increased cold exposure time. Sci. Rep..

[48] Treonis AM, Wall DH, Virginia RA (2000). The use of anhydrobiosis by soil nematodes in the Antarctic Dry Valleys. Funct Ecol.

[49] Yeates GW, Bongers T, Degoede RGM, Freckman DW, Georgieva SS (1993). Feeding-Habits in Soil Nematode Families and Genera - an Outline for Soil Ecologists. J Nematol.

[50] Hu SK (2016). Protistan diversity and activity inferred from RNA and DNA at a coastal ocean site in the eastern North Pacific. FEMS Microbiol Ecol.

[51] Hartmann M, Frey B, Mayer J, Mader P, Widmer F (2015). Distinct soil microbial diversity under long-term organic and conventional farming. ISME J.

[52] Lori Martina, Symnaczik Sarah, Mäder Paul, De Deyn Gerlinde, Gattinger Andreas (2017). Organic farming enhances soil microbial abundance and activity—A meta-analysis and meta-regression. PLOS ONE.

[53] Lupatini M, Korthals GW, de Hollander M, Janssens TKS, Kuramae EE (2017). Soil Microbiome Is More Heterogeneous in Organic Than in Conventional Farming System. Front Microbiol.

[54] Suleiman AKA (2018). Recycling organic residues in agriculture impacts soil-borne microbial community structure, function and N_2_O emissions. Sci Total Environ.

[55] Mäder P (2002). Soil fertility and biodiversity in organic farming. Science.

[56] Warren LA, Kendra KE, Brady AL, Slater GF (2016). Sulfur Biogeochemistry of an Oil Sands Composite Tailings Deposit. Front Microbiol.

[57] Martinez-Garcia LB, Korthals G, Brussaard L, Jorgensen HB, De Deyn GB (2018). Organic management and cover crop species steer soil microbial community structure and functionality along with soil organic matter properties. Agr Ecosyst Environ.

[58] Lupatini M, Korthals GW, Roesch LFW, Kuramae EE (2019). Long-term farming systems modulate multi-trophic responses. Sci Total Environ.

[59] Goldfarb KC (2011). Differential growth responses of soil bacterial taxa to carbon substrates of varying chemical recalcitrance. Front Microbiol.

[60] Saito T, Ishii S, Otsuka S, Nishiyama M, Senoo K (2008). Identification of Novel Betaproteobacteria in a Succinate-Assimilating Population in Denitrifying Rice Paddy Soil by Using Stable Isotope Probing. Microbes Environ.

[61] Yoshida M, Ishii S, Otsuka S, Senoo K (2009). Temporal shifts in diversity and quantity of nirS and nirK in a rice paddy field soil. Soil Biol Biochem.

[62] Orr CH, Stewart CJ, Leifert C, Cooper JM, Cummings SP (2015). Effect of crop management and sample year on abundance of soil bacterial communities in organic and conventional cropping systems. J Appl Microbiol.

[63] Francioli D (2016). Mineral vs. Organic Amendments: Microbial Community Structure, Activity and Abundance of Agriculturally Relevant Microbes Are Driven by Long-Term Fertilization Strategies. Front Microbiol.

[64] Lienhard P (2014). Pyrosequencing evidences the impact of cropping on soil bacterial and fungal diversity in Laos tropical grassland. Agron Sustain Dev.

[65] Floudas D (2015). Evolution of novel wood decay mechanisms in Agaricales revealed by the genome sequences of *Fistulina hepatica* and *Cylindrobasidium torrendii*. Fungal Genet Biol.

[66] Doveri F, Pecchia S, Vergara M, Sarrocco S, Vannacci G (2012). A comparative study of *Neogymnomyces virgineus*, a new keratinolytic species from dung, and its relationships with the Onygenales. Fungal Divers.

[67] Sugiyama Masato, Ohara Akiko, Mikawa Takashi (1999). Molecular phylogeny of onygenalean fungi based on small subunit ribosomal DNA (SSU rDNA) sequences. Mycoscience.

[68] Williams A, Manoharan L, Rosenstock NP, Olsson PA, Hedlund K (2017). Long-term agricultural fertilization alters arbuscular mycorrhizal fungal community composition and barley (*Hordeum vulgare*) mycorrhizal carbon and phosphorus exchange. New Phytol.

[69] Hodge A (2001). Arbuscular mycorrhizal fungi influence decomposition of, but not plant nutrient capture from, glycine patches in soil. New Phytol.

[70] Dai M (2014). Negative and positive contributions of arbuscular mycorrhizal fungal taxa to wheat production and nutrient uptake efficiency in organic and conventional systems in the Canadian prairie. Soil Biol Biochem.

[71] Treonis AM (2010). Effects of organic amendment and tillage on soil microorganisms and microfauna. Appl Soil Ecol.

[72] Xiong W (2018). Soil protist communities form a dynamic hub in the soil microbiome. ISME J..

[73] Yeates G (1987). Nematode feeding and activity: the importance of development stages. Biol Fert Soils.

[74] de Haan, J. *et al*. Biologische teelt op een zuidelijke zandgrond: opbrengst, bemesting, bodemkwaliteit en stikstofverliezen. ((Wageningen University & Research, Praktijkonderzoek AGV), 2018).

[75] de Haan, J. *et al*. Effect van organische stofbeheer op opbrengst, bodemkwaliteit en stikstofverliezen op een zuidelijke zandgrond. ((Wageningen University & Research, Praktijkonderzoek AGV), 2018).

[76] Quist CW (2016). Organic farming practices result in compositional shifts in nematode communities that exceed crop-related changes. Appl Soil Ecol.

[77] Schrama M, de Haan JJ, Kroonen M, Verstegen H, van der Putten WH (2018). Crop yield gap and stability in organic and conventional farming systems. Agr Ecosyst Environ.

[78] Wiesel L, Daniell TJ, King D, Neilson R (2015). Determination of the optimal soil sample size to accurately characterise nematode communities in soil. Soil Biol Biochem.

[79] Braid MD, Daniels LM, Kitts CL (2003). Removal of PCR inhibitors from soil DNA by chemical flocculation. J Microbiol Meth.

[80] Miller DN, Bryant JE, Madsen EL, Ghiorse WC (1999). Evaluation and optimization of DNA extraction and purification procedures for soil and sediment samples. Appl Environ Microb.

[81] de Hollander, M. NIOO-KNAW/hydra: Version 1.3. 3. Zenodo., 10.5281/zenodo.1434147 (2017).

[82] Köster J, Rahmann S (2012). Snakemake—a scalable bioinformatics workflow engine. Bioinformatics.

[83] Bushnell, B. BBMap: short read aligner and other bioinformatic tools v36.99, https://sourceforge.net/projects/bbmap/ (2018).

[84] Rognes T, Flouri T, Nichols B, Quince C, Mahé F (2016). VSEARCH: a versatile open source tool for metagenomics. PeerJ.

[85] Edgar RC, Haas BJ, Clemente JC, Quince C, Knight R (2011). UCHIME improves sensitivity and speed of chimera detection. Bioinformatics.

[86] Guillou L (2013). The Protist Ribosomal Reference database (PR2): a catalog of unicellular eukaryote Small Sub-Unit rRNA sequences with curated taxonomy. Nucleic Acids Res.

[87] Pruesse E, Peplies J, Glockner FO (2012). SINA: accurate high-throughput multiple sequence alignment of ribosomal RNA genes. Bioinformatics.

[88] Bokulich NA (2013). Quality-filtering vastly improves diversity estimates from Illumina amplicon sequencing. Nat Methods.

[89] Good IJ (1953). The population frequencies of species and the estimation of population parameters. Biometrika.

[90] Caporaso JG (2012). Ultra-high-throughput microbial community analysis on the Illumina HiSeq and MiSeq platforms. ISME J.

[91] Oksanen, J. *et al*. Vegan: community ecology package. R package version 2.3–0, https://CRAN.R-project.org/package=vegan (2015).

[92] Dhariwal A (2017). MicrobiomeAnalyst: a web-based tool for comprehensive statistical, visual and meta-analysis of microbiome data. Nucleic Acids Res.

[93] Segata N (2011). Metagenomic biomarker discovery and explanation. Genome Biol.

[94] Anderson M (2018). A lab-made method for extracting DNA from soils. Soil Res.

[95] Turner S, Pryer KM, Miao VP, Palmer JD (1999). Investigating deep phylogenetic relationships among cyanobacteria and plastids by small subunit rRNA sequence analysis 1. J Eukaryot Microbiol.

[96] Verbruggen E (2012). Testing Potential Effects of Maize Expressing the Bacillus thuringiensis Cry1Ab Endotoxin (Bt Maize) on Mycorrhizal Fungal Communities via DNA- and RNA-Based Pyrosequencing and Molecular Fingerprinting. Appl Environ Microb.

[97] Vainio EJ, Hantula J (2000). Direct analysis of wood-inhabiting fungi using denaturing gradient gel electrophoresis of amplified ribosomal DNA. Mycol Res.

[98] Lane, D. In *Nucleic Acid Techniques in Bacterial Systematics*. Vol. John Wiley &Sons 115–175 (Inc, 1991).

[99] Medlin L, Elwood HJ, Stickel S, Sogin ML (1988). The characterization of enzymatically amplified eukaryotic 16S-like rRNA-coding regions. Gene.

[100] Capra E (2016). A new primer set for DNA metabarcoding of soil Metazoa. Eur J Soil Biol.

