# Supplementary material for: Conventional and organic soil management as divergent drivers of resident and active fractions of major soil food web constituents
Source: Sci Rep. 2019 Sep 18;9:13521. doi: 10.1038/s41598-019-49854-y (PMC6751164; doi:10.1038/s41598-019-49854-y)
Supplement: Supplementary file 1 — Supplementary Tables and Figures [file 41598_2019_49854_MOESM1_ESM.pdf]

Supplementary information for:

Conventional and organic soil management as divergent drivers of resident and active fractions of major soil food web constituents

Paula Harkes[1^], Afnan K.A. Suleiman[2^], Sven J.J. van den Elsen[1], Janjo J. de Haan[3], Martijn Holterman[1], Eiko E. Kuramae[2], Johannes Helder[1*]

[1]P. Harkes, S.J.J. van den Elsen, M. Holterman, J. Helder (Hans.Helder@wur.nl). Laboratory of Nematology, Dept. Plant Sciences, Wageningen University, Droevendaalsesteeg 1, 6708 PB, Wageningen, the Netherlands. – [2]A.K.A. Suleiman, E.E. Kuramae NIOO-KNAW, Dept. Microbial Ecology, Droevendaalsesteeg 10, 6708 PB Wageningen, the Netherlands. – [3]J.J. de Haan, Wageningen University & Research Open Teelten, Edelhertweg 10, Lelystad, the Netherlands.

* Corresponding author. E-mail: Hans.Helder@wur.nl

^ These authors contributed equally to this work

Suppl. Table S1: Barley sampling dates of the two locations

	Vredepeel – ConMin, ConSlu, Org	Valthermond – Compost, Control
**Vegetative stage**	7th of June 2017	14th of June 2017
**Generative stage**	10th of July 2017	13th of July 2017

Suppl. Table S2: General run statistics of MiSeq.

	Forward reads	Reverse reads
**Average reads/sample**	74,694	74,694
**Total reads**	15,536,444	15,536,444
**Average bases/sample**	17,488,215	17,754,199
**Total bases**	3,637,548,813	3,692,873,434
**% bases Phred score > 30 (p<0.001)**	89.6%	76.1%

Suppl. Table S4: Results of PERMANOVA based on UniFrac distances (weighted and unweighted). The following variables were analyzed: Nucleic Acid (cDNA/DNA), Location (Vredepeel/Valthermond), Treatment (ConSlu, ConMin, Org (Vredepeel), Comp and No-Comp (Valthermond)), Sample type (Bulk/Rhizosphere) and Time point (Vegetative/Generative). Differences are considered significant if P <0.05.

**Bacteria**	**Unweighted UNIFRAC**		**weighted UNIFRAC**	
	R2	p	R2	p
Nucleic Acid	0.199	0.000	0.312	0.000
Location	0.116	0.000	0.169	0.000
Treatment	0.045	0.000	0.056	0.000
Sample Type	0.022	0.000	0.082	0.000
Time Point	0.011	0.006	0.007	0.014
Residuals	0.606		0.375	
**Fungi**				
Nucleic Acid	0.068	0.000	0.108	0.000
Location	0.078	0.000	0.089	0.000
Treatment	0.067	0.000	0.112	0.000
Sample Type	0.008	0.069	0.022	0.000
Time Point	0.015	0.006	0.019	0.000
Residuals	0.763		0.650	
**Protozoa**				
Nucleic Acid	0.184	0.000	0.142	0.000
Location	0.020	0.000	0.019	0.000
Treatment	0.019	0.015	0.017	0.097
Sample Type	0.008	0.022	0.013	0.003
Time Point	0.020	0.000	0.036	0.000
Residuals	0.748		0.773	
**Metazoa**				
Nucleic Acid	0.134	0.000	0.126	0.000
Location	0.034	0.000	0.085	0.000
Treatment	0.028	0.006	0.058	0.000
Sample Type	0.022	0.000	0.047	0.000
Time Point	0.008	0.084	0.012	0.006
Residuals	0.773		0.673	

Suppl. Table S5: Results of PERMANOVA testing samples of Valthermond for the effects of Nucleic Acid (cDNA and DNA), Treatment (compost and no compost), Sample Type (bulk soil and rhizosphere) and Time Point (vegetative and generative) on four soil-borne organismal groups.

Source	Df	SS	F	R2	P
**Bacteria**					
Nucleic Acid	1	0.164 66	34.089	0.43281	**9.999e-05**
Sample Type	1	0.01011	2.094	0.02659	0.09769 .
Treatment	1	0.00303	0.627	0.00796	0.56624
Time Point	1	0.00871	1.803	0.02289	0.13189
Residuals	28	0.13524		0.3555	
**Fungi**					
Nucleic_Acid	1	0.12438	8.1517	0.13886	**1.00E-04**
Sample_Type	1	0.01564	1.025	0.01746	**0.006099**
Treatment	1	0.04605	3.018	0.05141	0.374263
Time_Point	1	0.12438	8.1517	0.13886	**0.009499**
Residuals	31	0.47301		0.52808	
**Protozoa**					
Nucleic_Acid	1	0.3846	27.6554	0.27143	**1.00E-04**
Sample_Type	1	0.034	2.445	0.024	**0.05859**
Treatment	1	0.01579	1.1356	0.01115	0.27947
Time_Point	1	0.05514	3.9648	0.03891	**0.0121**
Residuals	44	0.61191		0.43185	
**Metazoa**					
Nucleic_Acid	1	0.38031	13.3288	0.21868	**1.00E-04**
Sample_Type	1	0.15642	5.482	0.08994	**1.00E-04**
Treatment	1	0.04597	1.6111	0.02643	0.10279
Time_Point	1	0.06569	2.3022	0.03777	**0.026**
Residuals	29	0.82746		0.47579	

Suppl. Table S6: Results of PERMANOVA analysis based on UniFrac distances (weighted and unweighted). The following variables were analyzed: Nucleic Acid (cDNA/DNA), Sample Type (bulk soil /rhizosphere), Treatment (soil management regime: ConMin, ConSlu, or Org), and Time Point (vegetative and generative). Differences are considered significant if P <0.05.

**Bacteria**	**Unweighted UNIFRAC**		**Weighted UNIFRAC**	
	R2	p	R2	p
Nucleic Acid	0.247	0.000	0.403	0.000
Treatment	0.062	0.000	0.083	0.000
Sample Type	0.027	0.000	0.100	0.000
Time Point	0.022	0.002	0.013	0.006
Residuals	0.643		0.401	
**Fungi**				
Nucleic Acid	0.085	0.000	0.148	0.000
Treatment	0.088	0.000	0.167	0.000
Sample Type	0.012	0.061	0.037	0.000
Time Point	0.035	0.001	0.022	0.000
Residuals	0.791		0.626	
**Protozoa**				
Nucleic Acid	0.217	0.000	0.149	0.000
Treatment	0.024	0.004	0.021	0.026
Sample Type	0.009	0.053	0.012	0.036
Time Point	0.035	0.000	0.060	0.000
Residuals	0.715		0.757	
**Metazoa**				
Nucleic Acid	0.168	0.000	0.141	0.000
Treatment	0.032	0.003	0.072	0.000
Sample Type	0.027	0.000	0.059	0.000
Time Point	0.023	0.001	0.024	0.000
Residuals	0.751		0.704	

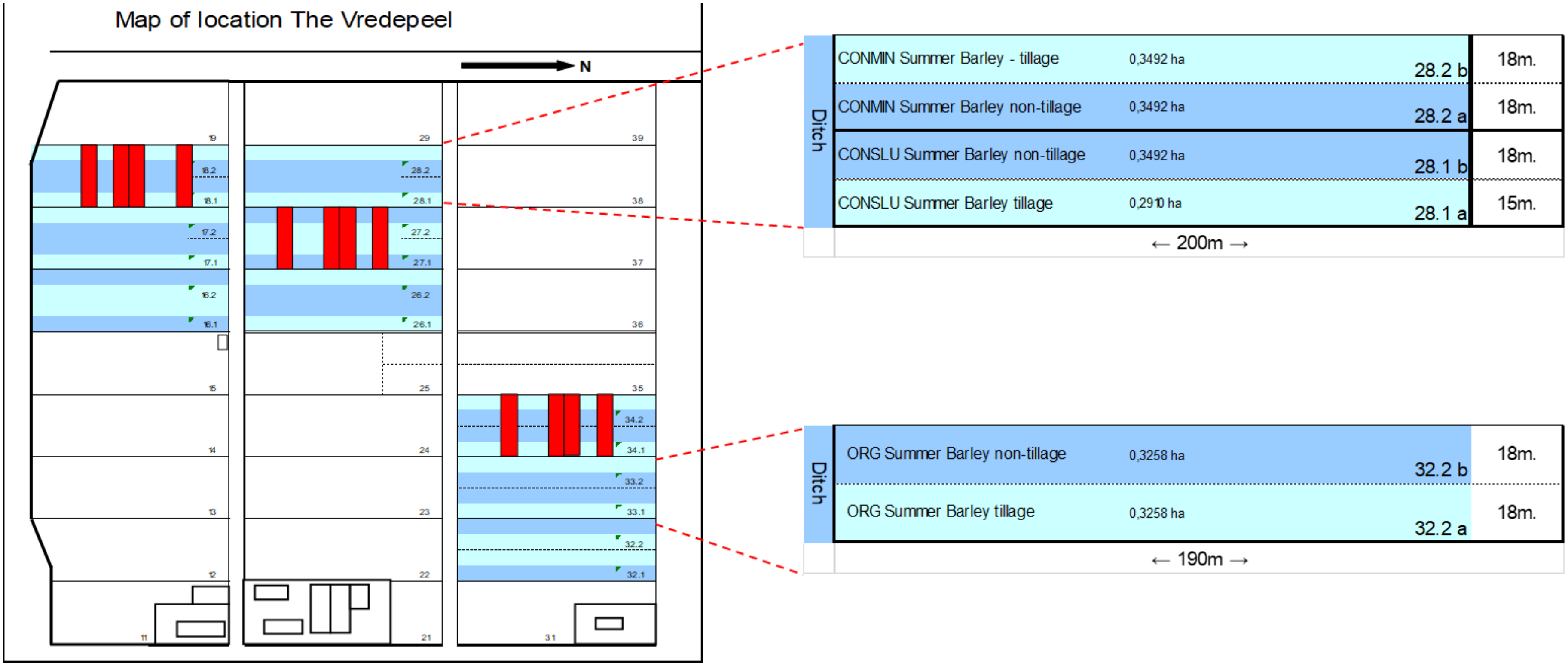

Figure S1: field set up location Vredepeel (sandy soils)

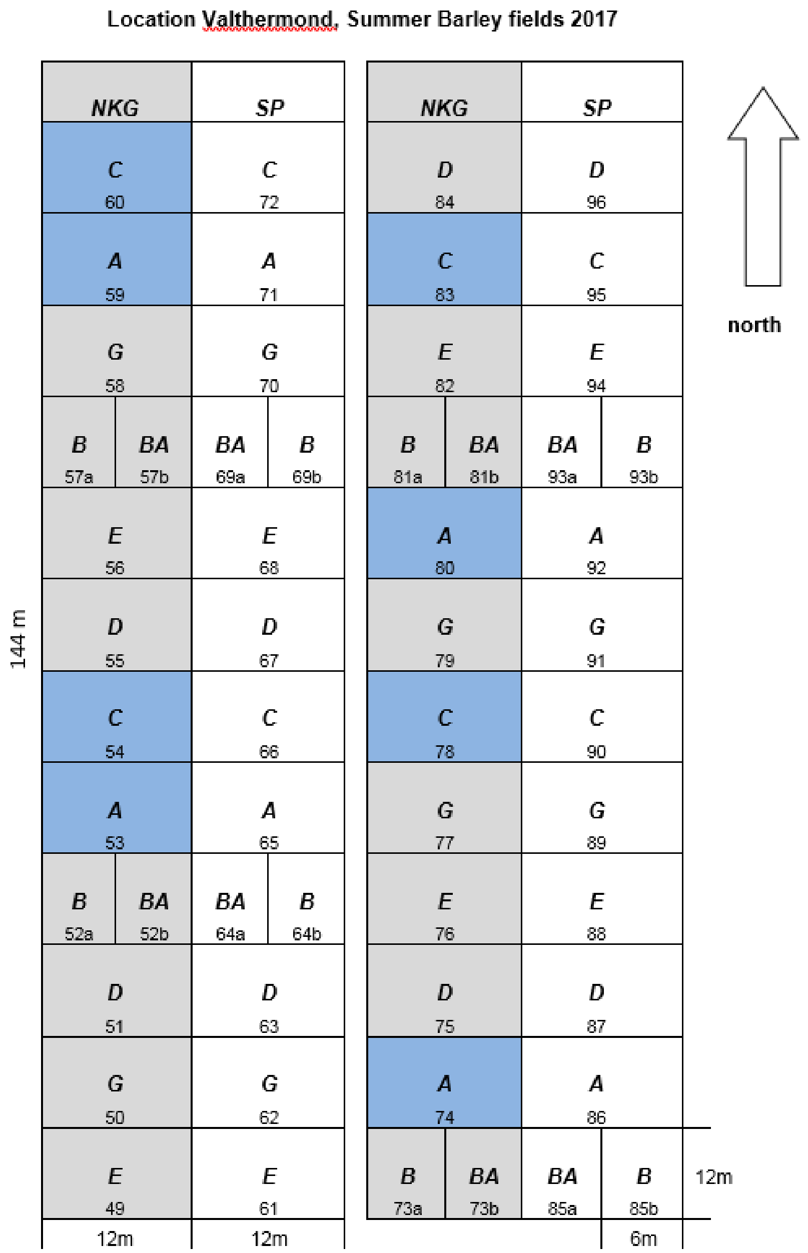

	Object
A	Standard
B	Tagetes
C	Compost
D	Hortinova bodembalance
E	Combination (BCDG - Targetes 1x / 8 years)
G	Rock flour
BA	Standaard (tagetes 1x / 8 years)
NKG	Non Tillage
SP	Tillage
SO	Sampled Object

Figure S2: field set up location Valthermond (peaty soils)

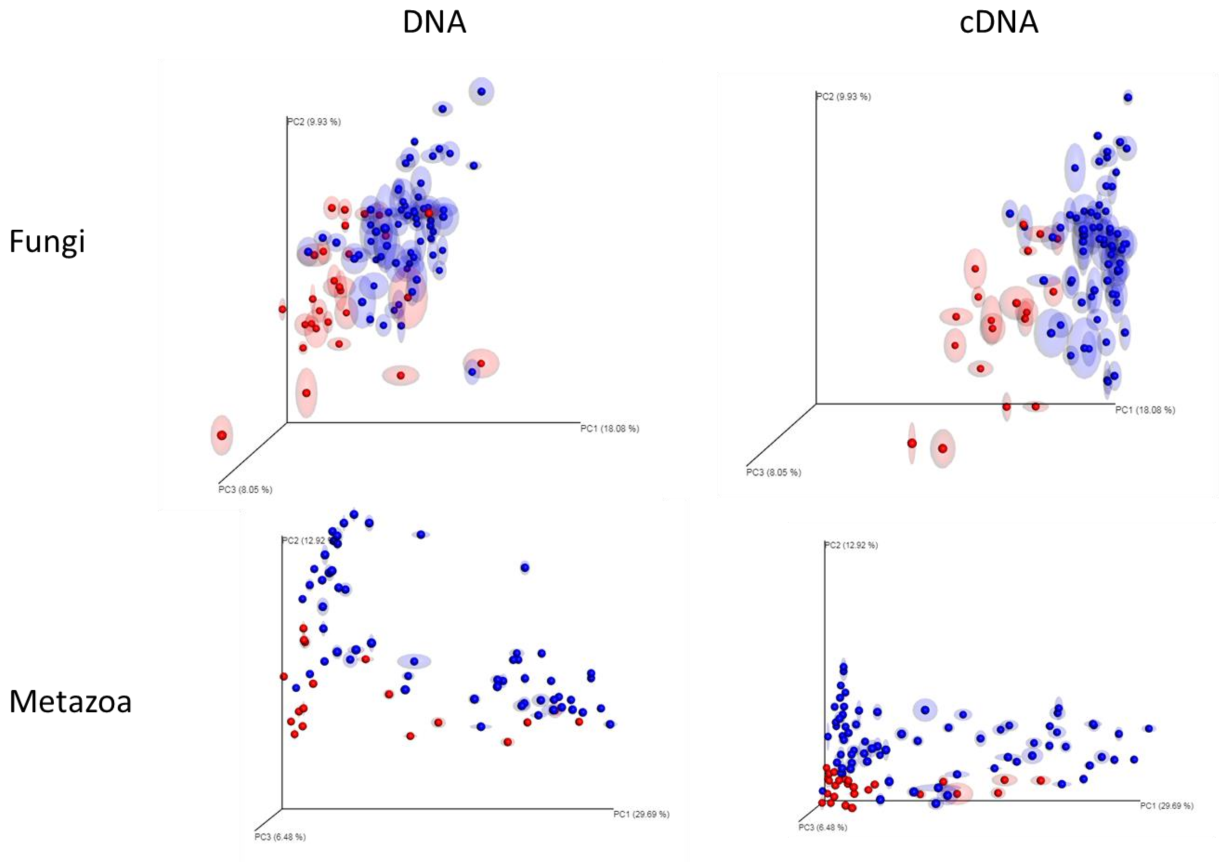

Figure S3: coordinate analysis (PCoA) plot with Bray-Curtis dissimilarity. Plots illustrating distances between communities in either DNA (n=104) A and C or cDNA (n=104) B and D. For Fungi A and B and Metazoa C and D. Distinguishing between Valthermond (red) and Vredepeel (blue).

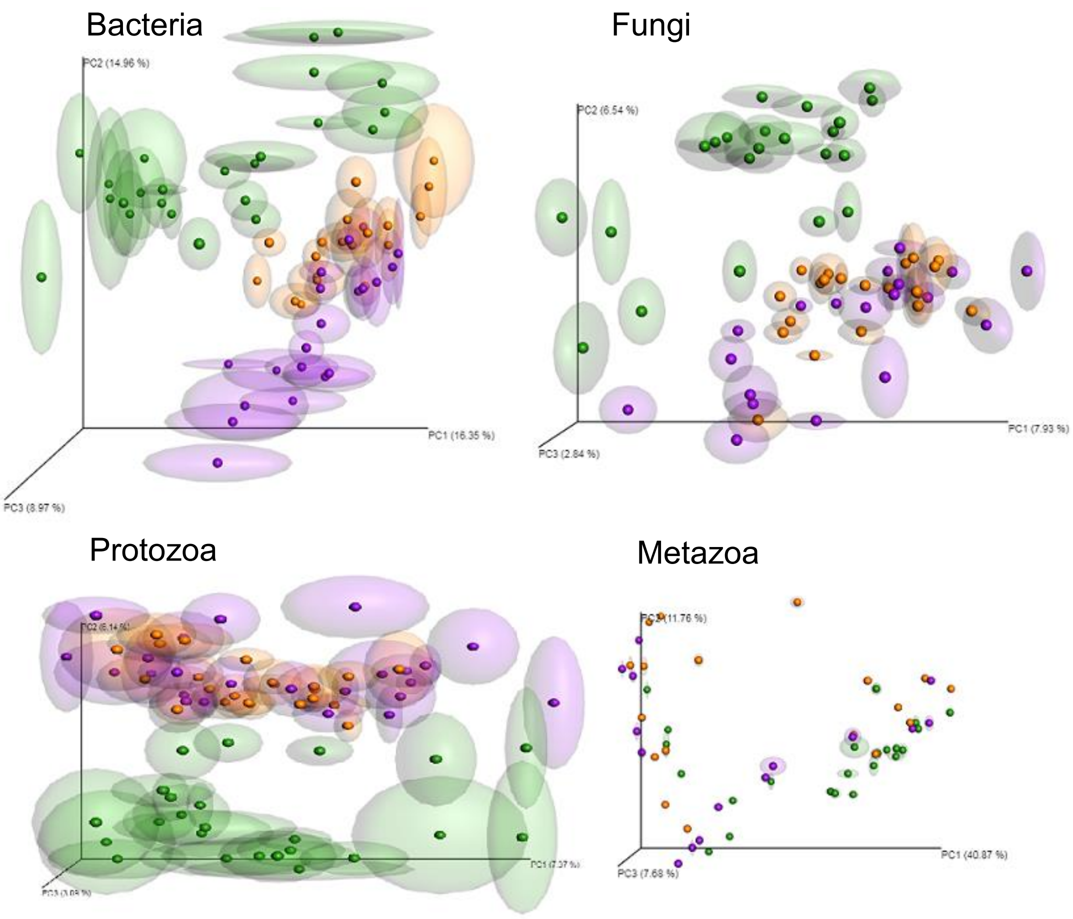

Figure S4: Principal coordinate analysis (PCoA) plot with Bray-Curtis dissimilarity. Plots illustrating distances between communities in all individual DNA samples from Vredepeel (n=72) for Bacteria, Fungi, Protozoa and Metazoa. Distinguishing between treatments: ConMin (purple) ConSlu (orange) and organic (green).

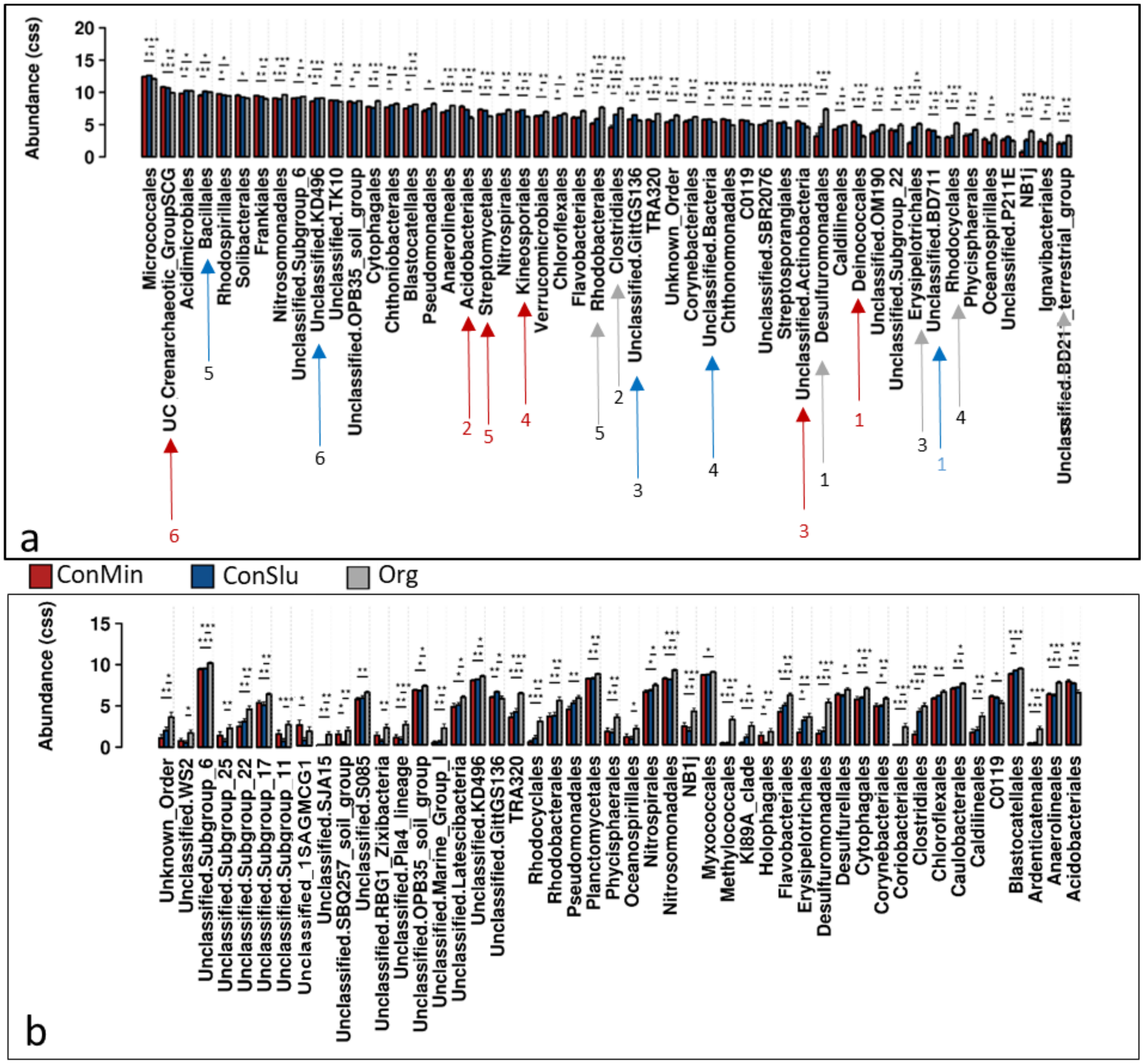

Figure S5.1: Differences in bacterial OTU abundance between the different soil management types (ConMin=red, ConSlu=blue and ORG=grey) for active (a) and total (b) bacterial communities. Asterisks represent statistically significant variance (ANOVA p-values; ∗p < 0.05, ∗∗p < 0.01, ∗∗∗p < 0.001) ANOVA. Arrows specify the order in which LefSe analysis categorises the indicative active orders.

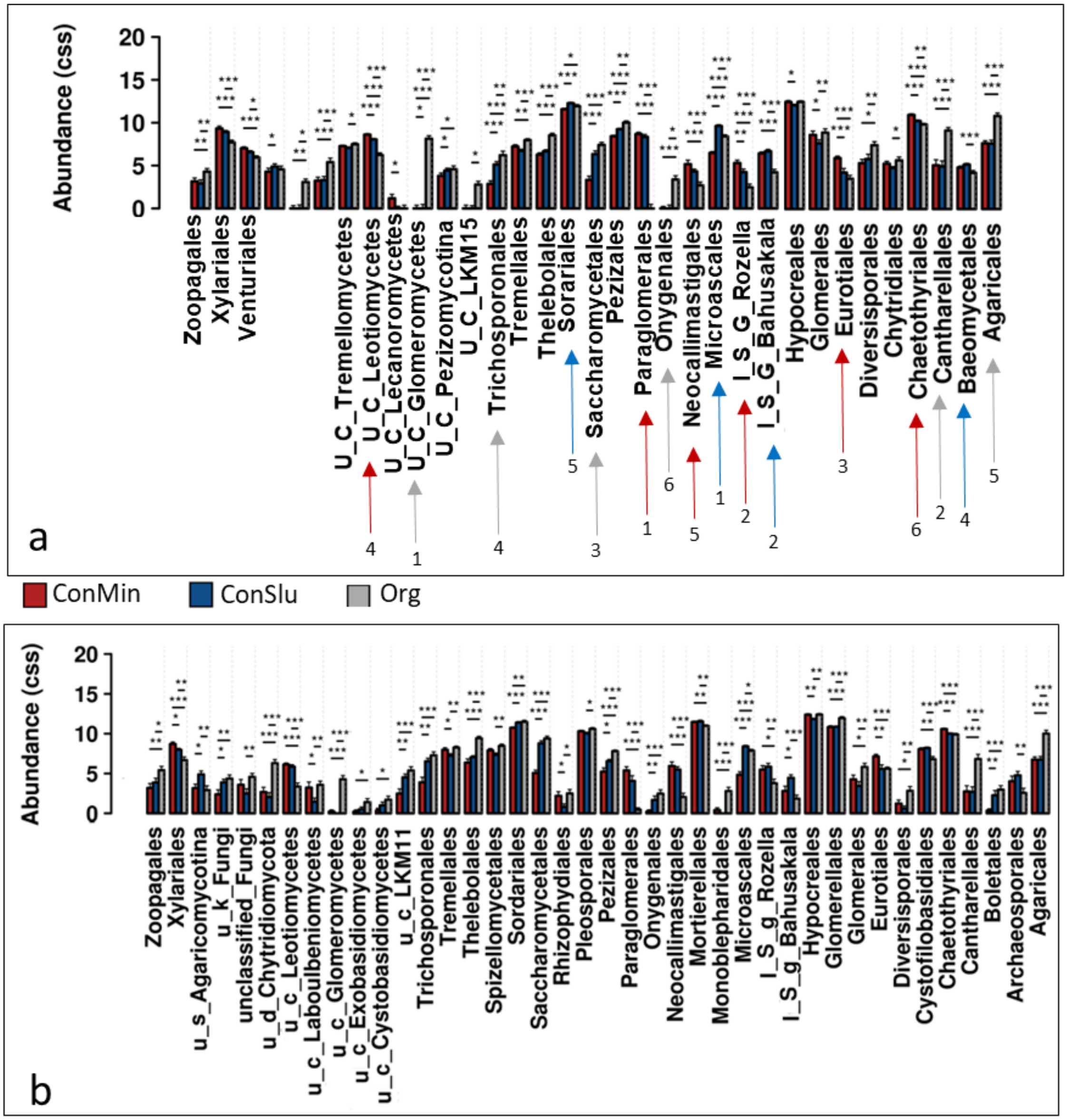

Figure S5.2: Differences in fungal OTU abundance between the different soil management types (ConMin=red, ConSlu=blue and ORG=grey) for active (a) and total (b) fungal communities. Asterisks represent statistically significant variance (ANOVA p-values; $*p < 0.05$, $**p < 0.01$, $***p < 0.001$) ANOVA. Arrows specify the order in which LefSe analysis categorises the indicative active orders.

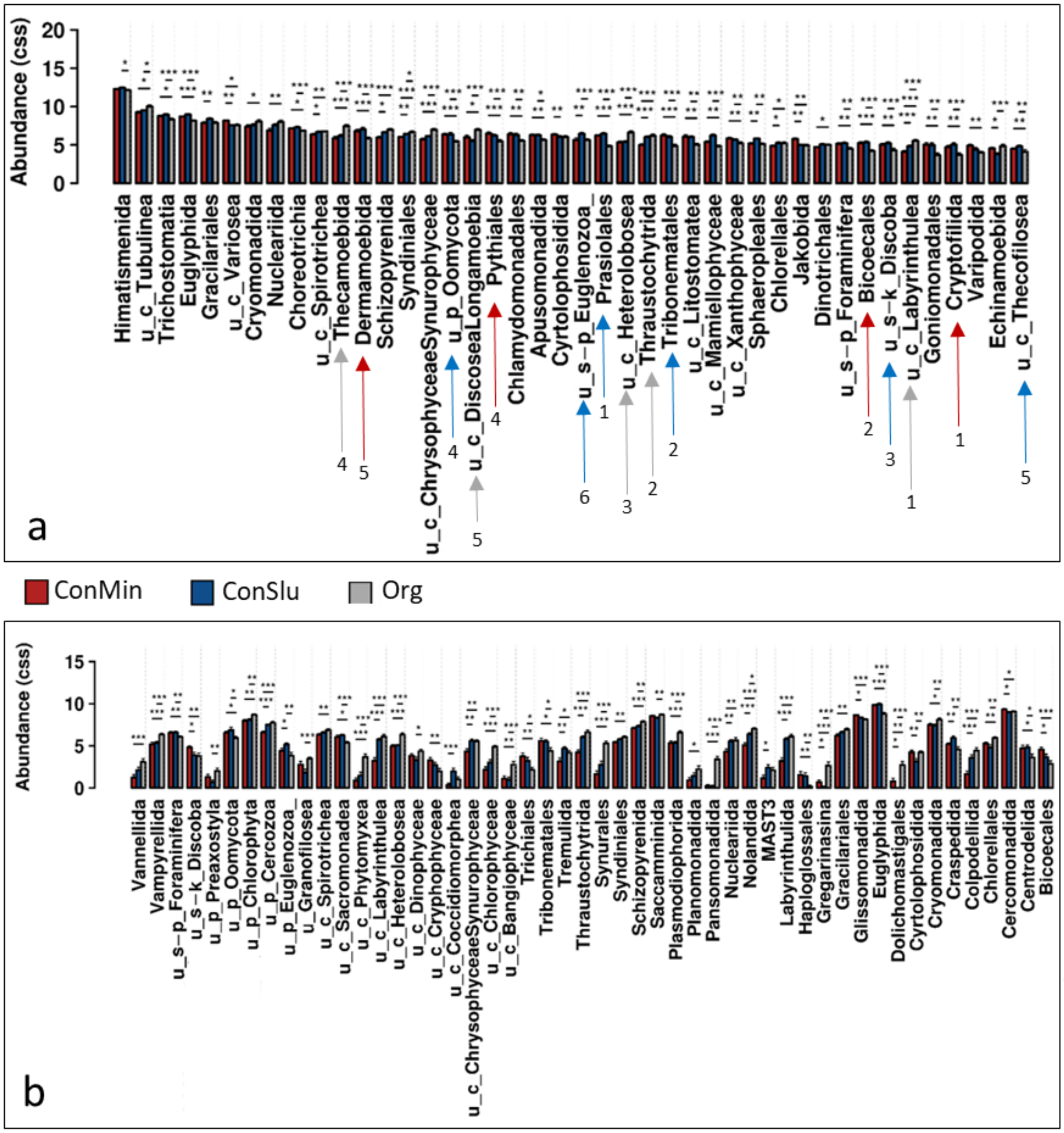

Figure S5.3: Differences in protist OTU abundance between the different soil management types (ConMin=red, ConSlu=blue and ORG=grey) for active (a) and total (b) protist communities. Asterisks represent statistically significant variance (ANOVA p-values; $*p < 0.05$, $**p < 0.01$, $***p < 0.001$) ANOVA. Arrows specify the order in which LefSe analysis categorises the indicative active orders.
